# PARENTAL TRANSITION TO LONG-TERM CARE: IMPLICATIONS FOR PSYCHOLOGICAL WELL-BEING OF ADULT CHILDREN

**DOI:** 10.1093/geroni/igad104.1879

**Published:** 2023-12-21

**Authors:** Viktoryia Kalesnikava, Annalise Lane, Linh Dang, Rodlescia Sneed, Briana Mezuk

**Affiliations:** University of Michigan, Ann Arbor, Michigan, United States; Department of Veterans Affairs, Ann Arbor, Michigan, United States; University of Michigan, Ann Arbor, Michigan, United States; Wayne State University, Detroit, Michigan, United States; University of Michigan, Ann Arbor, Michigan, United States

## Abstract

Approximately a quarter of US Baby Boomers provide informal caregiving for their parents. Though the psychological burden of caregiving is well-documented, the extent to which this burden relates to parental transition into long-term care is understudied. This study examined the longitudinal associations between parental transition to long-term care and life satisfaction and depressive symptoms among older adults of the ‘Baby Boom’ generation. Sample included respondents with at least one living parent from the Health and Retirement Study, 2010 - 2018 (n = 6,519, mean age = 56). Single-item life satisfaction was assessed on a 5-point Likert scale (range: not at all - completely satisfied). Depressive symptoms were indexed by the Composite International Diagnostic Interview (range: 0-8). Multivariate mixed effects poisson regressions of depressive symptoms and life satisfaction were fit, separately, adjusting for demographic characteristics, total wealth, and interview wave. Over the study period, 12% of respondents had a parent transitioning into long-term care. On average, respondents with parents transitioning into long-term care were older, wealthier, had higher depressive symptoms and lower life satisfaction. Longitudinally, parental transitions to long-term care was associated with mild increase in reported depressive symptoms, but there was no association with life satisfaction. Our findings are generally consistent with previous studies, reaffirming that while placement of parents in long-term care may alleviate caregiving burdens, it may not reduce distress in their adult children.

